# Protocol for Establishing a Stakeholder Group for Primary Care Research into Cancer Using a Modified 7P Framework and an e-Delphi Process

**DOI:** 10.12688/hrbopenres.13750.2

**Published:** 2023-12-19

**Authors:** Benjamin M. Jacob, Heather Burns, Ming Chuen Chong, Barbara Clyne, Primary Care PPI Group (HRB Primary Care Clinical Trials Network), Laura O'Connor, Kathleen Bennett, Patrick Redmond

**Affiliations:** 1 Department of General Practice, RCSI University of Medicine and Health Sciences, Dublin, Ireland; 2 National Cancer Control Programme, Health Service Executive, Dublin, Ireland; 3 Department of Public Health & Epidemiology, School of Population Health, RCSI University of Medicine and Health Sciences, Dublin, Ireland; 4 HRB Primary Care Clinical Trials Network, University of Galway, Galway, Ireland; 5 Data Science Centre, School of Population Heath, RCSI University of Medicine and Health Sciences, Dublin, Ireland

**Keywords:** Stakeholder engagement; Patient and public involvement; Steering group; Primary care; Cancer research; Research funding; Ireland

## Abstract

**Introduction:**

Currently, no group specifically supports and coordinates primary care focused cancer research in Ireland. The aim of this project is to establish an inclusive stakeholder group for primary care focused cancer research in Ireland, to coordinate research efforts and build capacity in researchers and institutions.

**Methods:**

We will convene a stakeholder group, recruiting individuals with personal and professional experience of cancer care in a community setting. “Core stakeholders”—patients, clinicians, researchers, and policymakers—will attend regularly. Additional “specialist stakeholders”, such as representatives of secondary care, private healthcare, health insurance, industry, cancer charities, and health research funders, will participate on an ad hoc basis. An e-Delphi consensus process will be used to assess the stakeholders’ views on: (1) the relevance and importance of primary care focused cancer research; (2) the potential role and scope of the stakeholder group; (3) how best to engage with lived experience stakeholders and healthcare professionals affected by the research; (4) how to encourage the dissemination of results and the translation of findings into practice. Round 1 will be open-ended and will invite the independent suggestions of stakeholders; in Round 2 and 3, group members will vote on the inclusion of these suggestions in a position statement by the group, with consensus defined as ≥75% agreement.

**Discussion:**

The formation of a broad stakeholder group to support primary care focused cancer research will ensure research is relevant, patient centered, and more readily translated into practice. It is also hoped that the group will support capacity building and strategic planning in this important research space.

## Introduction

### Background

Cancer imposes a significant healthcare burden in Ireland with an average of 35,825 invasive cancers diagnosed annually between 2018-2020 ^1^, costing €207 per capita or 5% of all healthcare expenditure costs annually—in addition to €116 per capita in indirect costs, due to lost earnings ^2^. Internationally, cancer research is a major destination for research funding ^3^, receiving approximately 25% of all disease-specific funding in the UK between 2004 and 2018 ^4^. In Ireland, the Health Research Board (HRB) reported that €35.8 million out of a total €131.8 million in 2021 was awarded to cancer related trials ^5^.

In the United Kingdom, the National Cancer Research Institute was established to promote collaboration between cancer research funders to maximise the value and benefits of cancer research for patients and the public ^6^. In Ireland, the National Cancer Control Programme (NCCP) has established a National Cancer Research Group which provides strategic leadership to the NCCP, Department of Health and other partners to implement the National Cancer Strategy ^7^. Similarly, the All-Island Cancer Research Institute provides a cancer research framework from “discovery to clinical implementation” in and between the Republic of Ireland and Northern Ireland ^8^.

The Irish Department of Health’s National Cancer Strategy (2017–2026) recommends that “clinical cancer research, and the staff who deliver it, become a fully integrated component of cancer care delivery” ^7^. Primary care professionals have a key role across the continuum of cancer care—in prevention, screening, early detection, treatment, survivorship and end of life care ^9– 11^. General Practitioners (GPs) are the initial point of contact for most patients presenting with symptoms of cancer ^12^. The delivery of primary care across the cancer continuum is influenced by unique structural and systems factors ^13, 14^. This requires specific study designs, methods, and funding to address primary care policy, practice, service, workforce design, and intervention development and evaluation ^15^. The research skillset and infrastructure they demand, and the relative underfunding of primary care research (vis-à-vis other areas of health services research) ^15– 19^, foster a research ecosystem with unique challenges and priorities.

However, in contrast to basic science and secondary care research, currently no group specifically supports and coordinates primary care focused cancer research in Ireland. This represents an important gap in the Irish research ecosystem because: (1) primary care shares a large part of the cancer control burden in any health system; (2) research is needed to inform and improve care; and (3) research findings from secondary care setting are typically not generalisable to primary care.

We believe that, to address these primary care-specific challenges and priorities, a stakeholder group which specifically supports and coordinates primary care focused cancer research is needed.

### Aims and objectives

1. Convening a stakeholder group

This protocol describes the establishment of an inclusive stakeholder group to support primary care focused cancer-related research in Ireland. This group will aim to address three broad needs: (1) to serve as a forum for key stakeholders, including the public, patients, clinicians, funders, researchers and policymakers to collaborate, enabling strategic planning and preventing duplication of research; (2) to build capacity in primary care cancer researchers, equipping them with knowledge and strengthening competence; (3) to improve research impact through strategic dissemination of research findings to clinicians and policymakers.

2. e-Delphi exercise

We will also conduct an e-Delphi exercise to gain consensus from stakeholders on: (1) the relevance and importance of primary care focused cancer research; (2) the potential role and scope of the stakeholder group; (3) how best to engage with lived experience stakeholders and healthcare professionals affected by the research; (4) how to encourage the dissemination of results and the translation of findings into practice.

## Methods

We will convene a stakeholder group, guided by the established recommendations for multistakeholder engagement in health research ^20^. Then we will conduct an e-Delphi exercise, in accordance with the Guidance on Conducting and REporting DElphi Studies (CREDES) ^21^. Any deviations from the methodology described by this protocol will be reported, justified by a rationale and applied systematically.

### 1. Convening a stakeholder group for primary care focused cancer research in Ireland

***Eligibility and diversity of stakeholders***

To be eligible for inclusion in the stakeholder group for primary care focused cancer research in Ireland, an individual must have personal experience of cancer care in a community setting (i.e., as a patient or caregiver), or have clinical, research, policy or advocacy experience in this area.

To achieve the goal of an inclusive membership, Concannon’s 7P taxonomy for stakeholder engagement will be utilised to ensure broad stakeholder representation and adapted to the Irish primary care context ^20^. The categories “Patients/Public”, “Providers”, “Principal Investigators (PIs)”, and “Policymakers” yield four “core stakeholder” groups which we have renamed: (1) “Patients”— Patient and Public Involvement (PPI) contributors, caregivers, and representatives from cancer advocacy organisations; (2) “Clinicians”—GPs and other healthcare professionals involved in cancer control in a community setting; (3) “Researchers”—academics and representatives of funding organisations with an interest in primary care focused cancer research; (4) “Policymakers”—HSE staff, civil servants or Department of Health representatives working in the area of primary care cancer policy.

To ensure that the perspectives of all key stakeholders of primary care cancer research are represented, we propose to recruit a minimum of two members from the four core stakeholder groups and recommend representation of all four at every meeting. However, the requirements for a quorum, as with all the operational methods, will be definitively determined by the group.

To facilitate the participation of additional stakeholders with more niche interests, we propose to distinguish between the core stakeholders described above, and “specialist stakeholders”, who are expected to contribute only when the meeting agenda concerns their area of expertise or interest. This distinction will serve to boost inclusivity by ensuring efficient stakeholder participation, enabling some stakeholders to decline meetings which are not relevant to their role, scope of practice or specialist interests.

**Figure 1.**
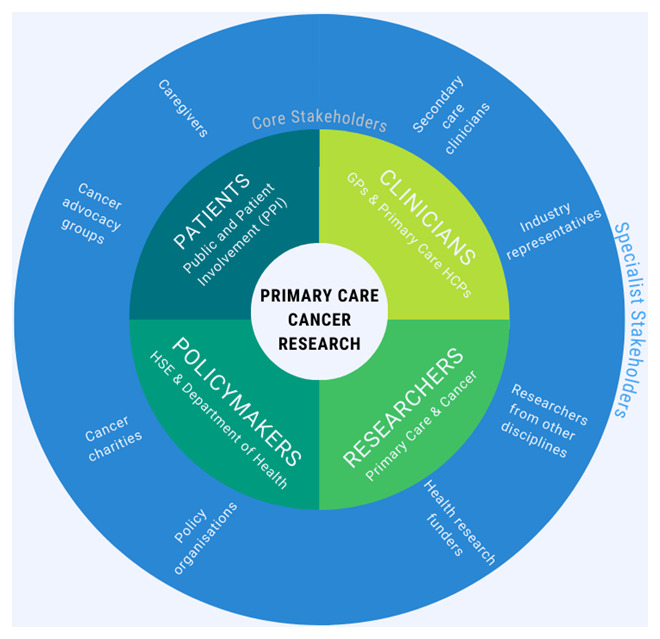
The stakeholders of primary care cancer research, and their role within the proposed group.

The three remaining categories of Concannon’s 7P taxonomy: “Purchasers” (of health insurance), “Payers” (i.e., health insurance companies), and “Product” (device and drug manufacturers), are more applicable to the US healthcare setting, and the activities of the stakeholder group may only occasionally be of relevance to them. Hence, we plan to invite representatives from private healthcare, health insurance, and industry (pharmaceutical companies, device manufacturers, and medical software) to participate on an ad-hoc basis as specialist stakeholders. The various stakeholders of primary care focused cancer research and their role within the proposed group is illustrated in Figure 1.

***Identification and recruitment***

We will adopt different recruitment strategies across the different stakeholder groups.

Patients and carers will be recruited via our Public and patient involvement (PPI) partners, the HRB PPI Ignite and HRB Primary Care Clinical Trials Network. Cancer advocacy organisations (the Irish Cancer Society, Marie Keating Foundation, and Breakthrough Cancer Research) will also be contacted to invite a representative to contribute to the stakeholder group.

Invites to clinicians and academics working in cancer care will be sent via established primary care and cancer research mailing lists and social media channels by the authors, who are involved with organisations such as the Association of University Department of General Practice in Ireland (AUDGPI), the HRB Primary Care Clinical Trials Network, the Irish College of General Practitioners (ICGP), and cancer research organisations. Policymakers and government representatives will be approached via the National Cancer Control Program and the National Cancer Registry.

***Operational methods of the stakeholder group***

The operational methods of the group will not be fixed in advance, but rather will be decided upon by the participating stakeholders, informed by the results of the e-Delphi exercise described below.

### 2. e-Delphi exercise

We will employ three rounds of an e-Delphi study to gather stakeholders’ beliefs, prior to the group’s first meeting, on four areas of interest:

(1) the relevance and importance of primary care focused cancer research,
(2) the potential role and scope of the stakeholder group,
(3) how best to engage with lived experience stakeholders and healthcare professionals affected by the research, and
(4) how to encourage the dissemination of results and the translation of findings into practice.

A Delphi process was chosen because it is a well-established methodology designed to systematically collate expert consultation and build consensus among a diverse group ^22, 23^. Additionally, the Delphi technique may be performed remotely, which will be convenient for the stakeholders. Stakeholder group members will be invited to participate in the e-Delphi exercise if they feel that they have an interest in primary care cancer research in general—i.e., all the core stakeholders and some of the specialist stakeholders.

***Software***

We will use Microsoft Forms to run the e-Delphi exercise. This will be piloted by the RCSI Department of General Practice staff and PPI contributors.

***Stages of the e-Delphi exercise***

Round 1 is open-ended; participants are free to make an unlimited number of suggestions in response to the following questions:

1. **Relevance**—Why is primary care cancer research important?
2(a). **Scope**
  i. What should be *included* within the scope of a stakeholder group for primary care cancer research?
  ii. What should be *excluded* from the scope of a stakeholder group for primary care cancer research?
2(b). **Role**—What kind of things should the stakeholder group do to further the cause of primary care cancer research in Ireland?
3. **Engagement**—How should a stakeholder group for primary care cancer research engage with those who have a lived experience of cancer?
4(a). **Dissemination**—What can this stakeholder group do to encourage the dissemination of findings?
4(b). **Impact**—What can this stakeholder group do to encourage the translation of research findings into benefits for patients?

In Round 2, the participants will be presented with the same 5 sections, but the task is voting (on the items generated from the previous round) rather than free-text answers. In sections 1 to 3, the participant will vote on inclusion in a finalised list to be published as a position statement. In sections 4 and 5, the participant will vote on whether a suggestion (a) should be included as a primary priority, (b) should be included as a secondary priority, or (c) should not be included at all in a finalised list to be published in the position statement. In Round 2, the participants are permitted to comment with their justification for any of the decisions/recommendations.

In Round 3, the participants will vote again on the same items as in Round 2, however this time they will be presented with both the results and justifications from Round 2.

After Round 3, if a consensus has not been achieved, the process will be terminated to prevent stakeholder fatigue, and a resolution will be sought at an in-person meeting of the stakeholder group. Both the failure to achieve consensus via the e-Delphi procedure, and the subsequent resolution achieved thereafter, will be reported.

The time between rounds is expected to be approximately one to two weeks, owing to the requirement to summarise the free-text answers from the previous round and to follow up with each participant who will be completing the exercise at a time of their choosing.

***Definition of consensus***

In sections 1 to 3 of the e-Delphi exercise, consensus is defined as ≥75% agreement that the item should be included or excluded ^24^. In sections 4 to 5 of the e-Delphi exercise, consensus is defined as ≥75% agreement that the item should be included in a given category. Any subsequent deviations from this definition of consensus will be justified and reported.

***Informational input and risk of bias***

Since Round 1 of the e-Delphi study is open-ended, no information will be provided to the participants so as not to bias them towards a particular answer or answers. After Round 1, the investigators will summarise the results of the open-ended round by merging and paraphrasing “suggestions” believed to possess the same meaning. This summarised list will be presented to the participants for Round 2 for voting—i.e., while the participants will vote on paraphrased suggestions from Round 1, they will be presented with the suggestions in their original form. After Round 2, the investigators will merge and paraphrase “justifications” believed to possess the same meaning. Again, in Round 3, participants will be presented with the justifications in both their paraphrased and original form.

## Discussion

### 1. Stakeholder group membership

We will describe the characteristics of stakeholder group members: their areas of interest, expertise, professional role and scope of practice, and institutional affiliations where appropriate, as well as their demographic characteristics. We will report separately the makeup of the e-Delphi “expert panel”, per the requirements of the CREDES reporting guideline.

### 2. e-Delphi results

We will outline the results of the e-Delphi exercise under the following headings:

(1) the relevance and importance of primary care focused cancer research,

(2) the potential role and scope of the stakeholder group,

(3) how best to engage with lived experience stakeholders and healthcare professionals affected by the research, and

(4) how best to disseminate results and advocate for translation of findings into practice.

We will report results for each round separately, to make transparent the evolving of consensus over successive rounds. This will include figures showing the average group response, changes between rounds, as well as any modifications of the survey instrument such as deletion, addition, or modification of survey items based on previous rounds.

### 3. Other results

At the first in-person meeting of the group, members will agree upon the operational methods of the group, a finalised list of objectives, and an action plan for the coming year, informed by the results of the e-Delphi exercise. This will form the basis of a “Terms of Reference” document for the group and will be reported as an appendix.

The stakeholder group will also develop a position statement on primary care focused cancer research in Ireland, informed by the results of the e-Delphi exercise – this will be included for publication (as an appendix). The position statement will comment on (1) the relevance and importance of primary care focused cancer research; (2) the potential role and scope of the stakeholder group; (3) how best to engage with lived experience stakeholders (the public, patients, and carers) and healthcare professionals affected by the research; (4) how best to disseminate results and advocate for translation of findings into practice.

We will include a reflection of potential limitations of the e-Delphi exercise and their impact on the resulting Terms of Reference and position statement of the stakeholder group ^21^. Where it is achieved, we will report endorsement of the position statement by relevant professionals and professional bodies to boost dissemination ^21^.

## Dissemination

In addition to a peer-reviewed open access publication and academic conference presentations, we will also disseminate the findings to a broad range of professional networks with relevance to primary care cancer research. Moreover, we intend to report on the progress of the project at a public event focusing on cancer control in primary care, planned for late 2023.

Once convened, the stakeholder will identify appropriate audiences to engage when sharing the outcomes, such as researchers, policymakers, funders, and patient and clinical communities.

## Conclusion

The formation of a broad stakeholder group in primary care focused cancer research, representing both professional and lived experience stakeholders, will ensure research is relevant, patient centred, and more readily translated into practice ^25^.

## Study status

At the time of submission, the stakeholder group had not yet been convened. It was planned that this would occur at during the period July to September 2023.

## Ethical considerations

The Research Ethics Committee at RCSI granted approval for this project (REC202301024)

## Data Availability

No data are associated with this article.
