# Supplementary material for: Protocol for Establishing a Stakeholder Group for Primary Care Research into Cancer Using a Modified 7P Framework and an e-Delphi Process
Source: HRB Open Res. 2023 Dec 19;6:53. Originally published 2023 Sep 13. [Version 2] doi: 10.12688/hrbopenres.13750.2 (PMC10822037; doi:10.12688/hrbopenres.13750.2)
Supplement: Peer review history

# Reviewer report: Reviewer response for version 2

**DOI:** 10.21956/hrbopenres.15154.r37515

**Published:** 2024-01-27

**Authors:** Jon Salsberg

**Affiliations:** 1 Primary Healthcare Research, University of Limerick, Limerick, County Limerick, Ireland

I am happy with the authors' responses to my comments and suggestions.

Is the study design appropriate for the research question?

Partly

Is the rationale for, and objectives of, the study clearly described?

Yes

Are sufficient details of the methods provided to allow replication by others?

Yes

Are the datasets clearly presented in a useable and accessible format?

Not applicable

Reviewer Expertise:

PPI, participatory health research, primary care research, systematic review, community and population health.

I confirm that I have read this submission and believe that I have an appropriate level of expertise to confirm that it is of an acceptable scientific standard.

# Reviewer report: Reviewer response for version 1

**DOI:** 10.21956/hrbopenres.15041.r36341

**Published:** 2023-10-18

**Authors:** Jon Salsberg

**Affiliations:** 1 Primary Healthcare Research, University of Limerick, Limerick, County Limerick, Ireland

I am very please to see this contribution to the literature. There is a need for guidance for and examples of stakeholder involvement as the imperative for PPI continues to grow.

 Overall, this is a good protocol which should help guide other groups, even beyond cancer care or primary care, to convene effective stakeholder groups.

 With this said, there are still a few points for improvement in the current manuscript.

 1) Protocol?

 1a) The title implies that the authors are presenting a novel protocol for establishing a stakeholder group. In fact, they are simply presenting Concannon et al's 2012 protocol.

 1b) The authors are appropriately using a Delphi process to assess and consolidate stakeholder views. However, there is nothing particularly novel about the approach or how they are using it.

 1c) Therefore, although this will be an interesting study, and I look forward to reading the outcomes, I'm not sure what we are gaining from a published protocol in advance of actual findings. However, SEE BELOW for some suggestions on how this could still be a valuable protocol.

 1d) Even if it does proceed, a better title would focus on 'Using an e-Delphi process to...'  Because that is what this is mostly about.

 2) Issues with the engagement approach

 2a) Although Concannon et al. is a good start and does indeed set the framework for a strong engagement process, it is getting a bit dated in terms of PPI scholarship (2012) and omits to address several issues that have emerged since.

 2a.i) Concannon does not directly address issues of representation, including EDI issues and reaching underrepresented or underserved segments of the population. Without this, steering groups tend to contain the 'usual suspects'. This would be an EXCELLENT opportunity for this protocol to ADD to Concannon and suggest something novel.

 2a.ii) Concannon does not directly address the ethical considerations surrounding steering group recruitment and selection. These are numerous but big ones are duality of relationships for PPI contributors (e.g., serving on the same panel as their health care provider and the power and vulnerabilities this can create); and researchers' duty of care toward vulnerable PPI contributors. Again, an excellent opportunity for the authors to ADD to Concannon.

Is the study design appropriate for the research question?

Partly

Is the rationale for, and objectives of, the study clearly described?

Yes

Are sufficient details of the methods provided to allow replication by others?

Yes

Are the datasets clearly presented in a useable and accessible format?

Not applicable

Reviewer Expertise:

PPI, participatory health research, primary care research, systematic review, community and population health.

I confirm that I have read this submission and believe that I have an appropriate level of expertise to confirm that it is of an acceptable scientific standard, however I have significant reservations, as outlined above.

# Response

**Published:** 2023-11-27

**Authors:** Benjamin Jacob

**Affiliations:** General Practice, Royal College of Surgeons in Ireland, 123 St Stephen's Green, Dublin 2, Ireland

**Comment/Suggestion #1:**

 "The title implies that the authors are presenting a novel protocol for establishing a stakeholder group. In fact, they are simply presenting Concannon et al's 2012 protocol... The authors are appropriately using a Delphi process to assess and consolidate stakeholder views. However, there is nothing particularly novel about the approach or how they are using it....Therefore, although this will be an interesting study, and I look forward to reading the outcomes, I'm not sure what we are gaining from a published protocol in advance of actual findings. However, SEE BELOW for some suggestions on how this could still be a valuable protocol... Even if it does proceed, a better title would focus on 'Using an e-Delphi process to...'  Because that is what this is mostly about."

**Response #1**:

 As you’ve already said, our approach to establishing the stakeholder group involves two aspects: (1) convening a stakeholder group, using a modified Concannon 7P Framework, and (2) exploring stakeholder values in order to guide the process, using an e-Delphi process. Therefore, it feels like the title should not the e-Delphi process without mentioning the 7P Framework also. 

 We could thus suggest a new title as follows: “Protocol for Establishing a Stakeholder Group for Primary Care Research into Cancer Using a Modified 7P Framework and an e-Delphi Process”. It might be considered a little longwinded? But if you find a shorter title—e.g., Protocol for Establishing SPARC—a Stakeholder group for Primary cAre Research into Cancer—to be misleading, we would be happy to accept the former. 

 Our rationale for publishing the protocol is not because of the novelty of the methods (as you rightly point out, we are following a well-established approach). Rather our primary motivation was academic transparency (and the consequent reduction of publication bias/selective reporting), as well as the reduction of research duplication. 

**Comment/Suggestion #2:**

 "Although Concannon et al. is a good start and does indeed set the framework for a strong engagement process, it is getting a bit dated in terms of PPI scholarship (2012) and omits to address several issues that have emerged since... Concannon does not directly address issues of representation, including EDI issues and reaching underrepresented or underserved segments of the population. Without this, steering groups tend to contain the 'usual suspects'. This would be an EXCELLENT opportunity for this protocol to ADD to Concannon and suggest something novel."

**Response #2**:

 We agree with the 2022 Declaration of Helsinki position that “groups that are underrepresented in medical research should be provided appropriate access to participation in research.” This is one of the research principles which a dedicated stakeholder group can support. 

 You are right to say that we have not proposed a clear EDI strategy in advance of establishing the group, but this is because we want the stakeholders themselves to decide upon the group’s responsibilities in accordance with their values (rather than having this enforced a priori by the research team). Hence, we expect the stakeholders to make suggestions along these lines via the e-Delphi exercise, due to the diversity of stakeholders and PPI input in the group. A natural place for these suggestions to be made is Q2b (Role of the group) and Q3 (Engagement with lived experience stakeholders). Thus, we can be reasonably confident that the outcome of the e-Delphi consensus process will ensure that EDI and PPI concerns are addressed, and an action plan published in the planned position statement. 

 This group will support and promote high-quality research, which involves the promotion of meaningful PPI input and the inclusion of under-represented and underserved groups in the population. However, this stakeholder group will not act as a steering group for any specific research projects. Futhermore, the under-represented and underserved groups in the population differ depending on the cancer type and clinical context, thus the stakeholder group will best effect positive change through its general influence of research steering committees and will seek a diversity of lay and professional voices in its composition.

**Comment/Suggestion #3:**

 "Concannon does not directly address the ethical considerations surrounding steering group recruitment and selection. These are numerous but big ones are duality of relationships for PPI contributors (e.g., serving on the same panel as their health care provider and the power and vulnerabilities this can create); and researchers' duty of care toward vulnerable PPI contributors. Again, an excellent opportunity for the authors to ADD to Concannon."

**Response #3**:

 These ethical concerns are less applicable to this kind of stakeholder group, as it is concerned with shaping overarching research priorities, capacity building and impact strategies rather than acting as a typical project steering committee (handling the nitty gritty details of study design and project management etc.). 

 For clarity, none of the PPI contributors in this stakeholder group will serve on the same panel as their health care provider (albeit this can occur in project steering committees). Secondly the researchers’ duty of care towards vulnerable PPI contributors doesn’t clearly apply because the “project” they are working on is not a “research study” being “led” by a “researcher”, and to which vulnerable patients, for example, are contributing in a PPI capacity. Rather, in the case of this stakeholder group, PPI contributors and researchers are just two of wider range of possible collaborators, working together to support primary care cancer research at a strategy level.

# Reviewer report: Reviewer response for version 1

**DOI:** 10.21956/hrbopenres.15041.r36148

**Published:** 2023-09-15

**Authors:** Desmond Leddin

**Affiliations:** 1 Department of Medicine, Dalhousie University, Halifax, Nova Scotia, Canada

Thank you for the opportunity to review the protocol, 

 The authors are very well qualified to carry out the work and are in an academic environment which will be supportive.

- Is the rationale for, and objectives of, the study clearly described?
  The rationale is clearly described. The administrative relationship of the proposed new group to the NCRG or All Island CRI is not clear. The authors mention that there are basic science and secondary care research groups. It would be of interest to know how they relate to the NCRG.
- Is the study design appropriate for the research question?
  It is. The authors propose to use a modified 7P structure, which is certainly permissible within the method described by Concannon.
- Are sufficient details of the methods provided to allow replication by others?
  - Yes. More detail on how the suggestions from Round 1 will be extracted for presentation in subsequent rounds would be helpful.
  - The aims seem to change a little as the process moves to methods.
    - Page 3. Under Aims 2. Edelphi four questions are listed. Relevance and importance, role and scope, engagement, dissemination, advocacy and translation into policy and practice. Arguably this is six questions.
    - Page 5 Edelphi exercise. Same as above
    - Page 5. Stages of the e Delphi exercise lists relevance, scope, role, engagement, and impact.
    - This is somewhat different from the previously listed aims. Benefits for patients may bring advocacy and translation back in and perhaps that is meant by impact? Impact was listed on page 3 under stakeholder objective but not under the eDelphi exercise.

Overall, a well written, and well-constructed proposal. My comments are minor. Thank you for allowing me to read it.

Is the study design appropriate for the research question?

Yes

Is the rationale for, and objectives of, the study clearly described?

Yes

Are sufficient details of the methods provided to allow replication by others?

Partly

Are the datasets clearly presented in a useable and accessible format?

Not applicable

Reviewer Expertise:

Colon cancer screening, environment and health

I confirm that I have read this submission and believe that I have an appropriate level of expertise to confirm that it is of an acceptable scientific standard.

# Response

**Published:** 2023-11-28

**Authors:** Benjamin Jacob

**Affiliations:** General Practice, Royal College of Surgeons in Ireland, 123 St Stephen's Green, Dublin 2, Ireland

**COMMENT #1**:

*"The aims seem to change a little as the process moves to methods.*

- *Page 3. Under Aims 2. Edelphi four questions are listed. Relevance and importance, role and scope, engagement, dissemination, advocacy and translation into policy and practice. Arguably this is six questions.*
- *Page 5 Edelphi exercise. Same as above*
- *Page 5. Stages of the e Delphi exercise lists relevance, scope, role, engagement, and impact.*
- *This is somewhat different from the previously listed aims. Benefits for patients may bring advocacy and translation back in and perhaps that is meant by impact? Impact was listed on page 3 under stakeholder objective but not under the eDelphi exercise.*"

**RESPONSE #1**:

 We have made slight alterations to the three sections for greater correspondence and clarity:

 Page 3 & 5 ("2. e-Delphi exercise") both now read:

 “(1) the relevance and importance of primary care focused cancer research; (2) the potential role and scope of the stakeholder group; (3) how best to engage with lived experience stakeholders and healthcare professionals affected by the research; (4) how to encourage the dissemination of results and the translation of findings into practice.”

 Page 5 ("Stages of the e-Delphi exercise") now has sub-questions (designated 2(a), 2(b), 4(a) and 4(b)) so as to enable consistency between the numbering schemes in each instance:

 “1. Relevance—Why is primary care cancer research important?

 2(a). Scope 

 i. What should be included within the scope of a stakeholder group for primary care cancer research?

 ii. What should be excluded from the scope of a stakeholder group for primary care cancer research?

 2(b). Role—What kind of things should the stakeholder group do to further the cause of primary care cancer research in Ireland?

 3. Engagement—How should a stakeholder group for primary care cancer research engage with those who have a lived experience of cancer?

 4(a). Dissemination—What can this stakeholder group do to encourage the dissemination of findings?

 4(b). Impact—What can this stakeholder group do to encourage the translation of research findings into benefits for patients?”
